# *LipidOz* enables automated elucidation of lipid carbon–carbon double bond positions from ozone-induced dissociation mass spectrometry data

**DOI:** 10.1038/s42004-023-00867-9

**Published:** 2023-04-19

**Authors:** Dylan H. Ross, Joon-Yong Lee, Aivett Bilbao, Daniel J. Orton, Josie G. Eder, Meagan C. Burnet, Brooke L. Deatherage Kaiser, Jennifer E. Kyle, Xueyun Zheng

**Affiliations:** 1 Pacific Northwest National Laboratory, Richland, WA 99354 USA; 2 Present Address: PrognomiQ, Inc, San Mateo, CA 94403 USA

**Keywords:** Lipidomics, Bioanalytical chemistry, Mass spectrometry, Cheminformatics, Computational chemistry

## Abstract

Lipids play essential roles in many biological processes and disease pathology, but unambiguous identification of lipids is complicated by the presence of multiple isomeric species differing by fatty acyl chain length, stereospecifically numbered (sn) position, and position/stereochemistry of double bonds. Conventional liquid chromatography-mass spectrometry (LC-MS/MS) analyses enable the determination of fatty acyl chain lengths (and in some cases sn position) and number of double bonds, but not carbon-carbon double bond positions. Ozone-induced dissociation (OzID) is a gas-phase oxidation reaction that produces characteristic fragments from lipids containing double bonds. OzID can be incorporated into ion mobility spectrometry (IMS)-MS instruments for the structural characterization of lipids, including additional isomer separation and confident assignment of double bond positions. The complexity and repetitive nature of OzID data analysis and lack of software tool support have limited the application of OzID for routine lipidomics studies. Here, we present an open-source Python tool, *LipidOz*, for the automated determination of lipid double bond positions from OzID-IMS-MS data, which employs a combination of traditional automation and deep learning approaches. Our results demonstrate the ability of *LipidOz* to robustly assign double bond positions for lipid standard mixtures and complex lipid extracts, enabling practical application of OzID for future lipidomics.

## Abstract (web summary)

Ozone-induced dissociation (OzID) coupled with ion mobility spectrometry-mass spectrometry (IMS-MS) provides the capacity for in-depth structural elucidation of lipids with isomer separation and confident assignment of double bond positions, however, OzID data analysis remains very challenging. Here, the authors develop a Python tool, LipidOz, for the automated determination of lipid double bond locations from complex LC-OzID-IMS-MS data, with a combination of traditional automation and deep learning approaches.

## Introduction

Lipids play important roles in the formation of cellular structure^1^ and take part in complex signaling as part of both homeostatic processes and disease pathology^2^. Lipidomics, the study of all lipids in a biological sample, is an important approach for elucidating insight into complex biological processes. A critical component of lipidomics analyses is lipid identification, which can be performed at multiple levels of structural detail (i.e. including information about lipid class, fatty acid composition, etc.), each having implications on the biological interpretations and underlying mechanisms^3^. Advances in analytical technologies in recent years, including increased adoption of high-resolution mass spectrometry (HRMS)^4^, have increased the level of structural detail that can be routinely achieved for lipid identifications in lipidomics studies. However, the assignment of double bond positions within lipids is not possible using most conventional analyses. Lipid double bond positions are an important structural characteristic, with the double bond position determining whether signaling molecules derived from the oxidation of fatty acids mediate pro- or anti-inflammatory responses^2^. and differentiating breast cancer cell lines^5^.

**Fig. 1:**
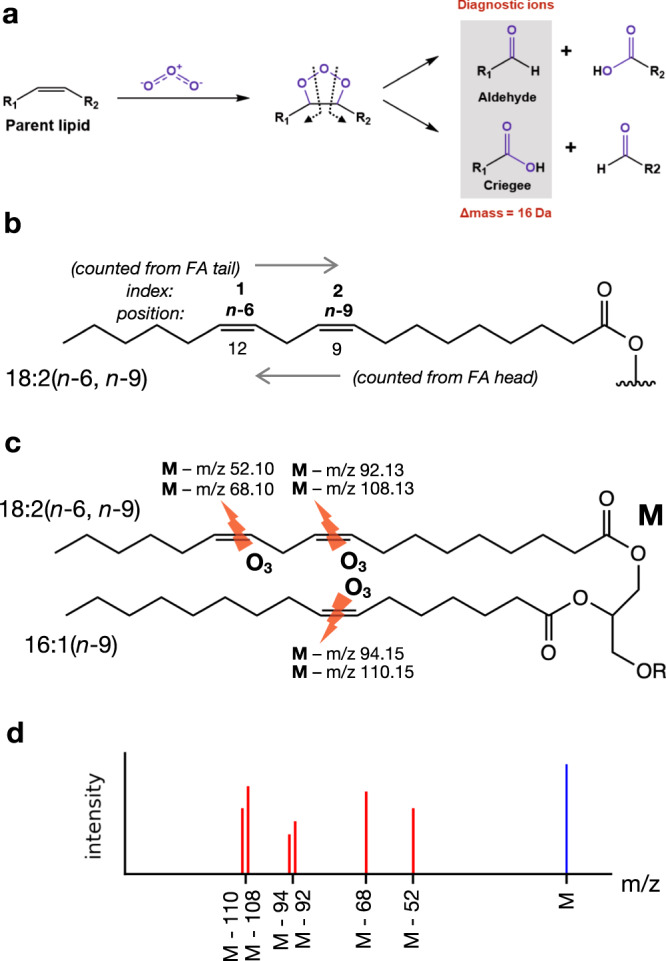
Schematic of OzID reaction chemistry for lipid double bond determination. **a** Ozone molecule reacts with carbon–carbon double bonds in unsaturated lipids, resulting pairs of characteristic fragment ions differing in mass by 16 Da. **b** The nomenclature for C=C double bond locations: the position(s) of unsaturation is indicated to be x carbons from the methyl end of the acyl chain with the nomenclature (*n*-x), multiple double bonds in polyunsaturated lipids are indicated with index number counting from the methyl end of fatty acyl chain. **c**, **d** OzID produces pairs of fragment ions (as neutral losses from the precursor m/z, M) with masses that allow assignment of double bond positions and indices.

Ozone-induced dissociation (OzID) or ozonolysis is an effective means of identifying double bond positions in unsaturated lipids^6–8^. OzID leverages a gas-phase oxidation reaction between a carbon–carbon double bond within an unsaturated lipid molecule and the ozone molecule, resulting in cleavage of the double bond and yielding a characteristic pair of fragment ions called aldehyde and criegee ions with a mass difference of 16 Da, which can be used for unambiguous determination of double bond positions in lipids (Fig. 1a). Depending on the position and index of the double bond along the fatty acyl chain (Fig. 1b), OzID at each double bond in a lipid precursor ion will result in a pair of fragment ions as neutral losses with masses that are diagnostic for the double bond position and index (Fig. 1c). Double bond positions (and indices) can therefore be assigned for a lipid precursor by examining the mass spectrum for all such pairs of diagnostic fragments (Fig. 1d). Given the success of OzID, the technique has been incorporated with different mass spectrometry platforms. More recently, OzID has been shown to perform efficiently in timescales that are compatible with ion mobility spectrometry (IMS) separations without sacrificing throughput^8^. In addition to the benefit of isomer separation capability from IMS^9,10^, the high-pressure feature in IMS instrument also enables a greater reaction efficiency for OzID and therefore results in fragment ions with a much higher abundance for confident identification. Thus, multi-dimensional analyses incorporating liquid chromatography, OzID and IMS-MS (LC-OzID-IMS-MS) can be used to generate structurally rich lipidomics data that allows in-depth identification of lipids that includes separation of isomers and assignment of double bond positions^8–11^.

One challenge that has been limiting the broad application of OzID for routine lipidomics studies comes from the complexity of the data analysis. The data analysis is currently manual and involves several steps of data extraction and processing which must be repeated for dozens of putative OzID fragments per lipid precursor, making interpretation of the results highly time- and labor-intensive. Thus, there is a need for informatics tools that streamline the analysis of OzID data in order to perform data analysis in an automated and higher throughput manner. To address the gap and accelerate the structural elucidation of lipids, we developed *LipidOz*: a Python tool for the automated identification of lipid double bond positions from complex LC-OzID-IMS-MS data using a combination of traditional automation and deep learning (DL) approaches. In this work, we demonstrate the ability of the *LipidOz* tool to robustly assign lipid double bond positions for lipid standards and complex tissue lipid extracts.

## Results

### Overview of OzID data analysis workflow for determining lipid double bond positions

**Fig. 2:**
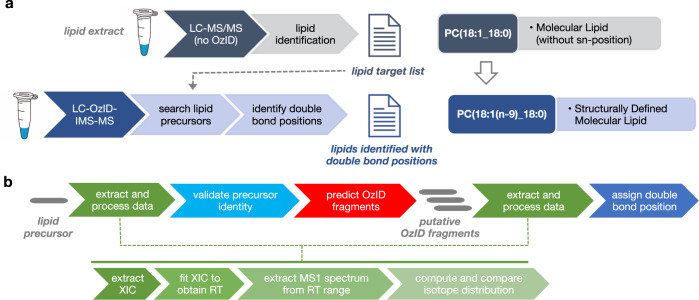
OzID data acquisition and analysis workflows. **a** Schematic representation of lipidomics workflow. Initial lipid identifications are made at the molecular lipid level from LC-MS/MS analysis. These initial identifications are used as a target list to search for lipid precursors and identify double bond positions from the LC-OzID-IMS-MS data, yielding identifications at the structurally defined molecular lipid level. **b** Schematic representation of OzID data extraction and processing. First, raw data is extracted and processed for a lipid precursor, which involves extracting and fitting XICs and MS1 spectra to obtain observed retention time and isotope distributions. This information is used to validate the identity of the precursor, then all possible OzID fragments are predicted using the fatty acid composition of the precursor. For each of these putative OzID fragments, raw data is extracted and processed in the same fashion as for the precursor, then double bond position(s) may be assigned based on the extracted chromatographic and mass spectral information.

The pipeline for structure elucidation of lipids including the double bond positions is shown in Fig. 2 and individual steps are described in detail in the later sections. Briefly, identification of lipid double bond positions is achieved in two steps (Fig. 2a). First, initial lipid identifications are obtained and validated from traditional liquid chromatography-tandem mass spectrometry (LC-MS/MS) data^12^, where the lipid class identity and the fatty acyl composition of the lipids are confirmed and an associated target list, containing initial lipid identifications and corresponding retention times, is constructed. Next, this target list is used to identify lipids for double bond assignment from the LC-OzID-IMS-MS data. The individual data analysis steps are described in detail in the “Methods” sections, but briefly, this the process of identifying the double bond position consists of iterative data extraction and processing to validate precursor identity and assign diagnostic OzID fragments that can be used to assign double bond positions (Fig. 2B). For each lipid precursor ion, an extracted ion chromatogram (XIC) is extracted using the precursor *m*/z and fit to obtain a retention time. This retention time is then used to extract a mass spectrum including the M, M + 1, and M + 2 isotopes, from which the identity of the precursor ion can be verified based on agreement between observed and predicted isotope abundances. Then, all possible double bond positions and their corresponding pairs of OzID fragment ions are predicted based on the composition of the fatty acids in the precursor ion. For each pair of putative OzID fragment ions, XICs and mass spectra are extracted and processed in a similar fashion to the precursor ion. The carbon–carbon double bond positions are assigned based on agreement between observed and theoretical isotope distributions for the corresponding diagnostic OzID fragment pairs, in addition to agreement between their retention times and that of the precursor ion. This process is repeated for all precursor ions in the target list.

### Automated OzID data analysis

**Fig. 3:**
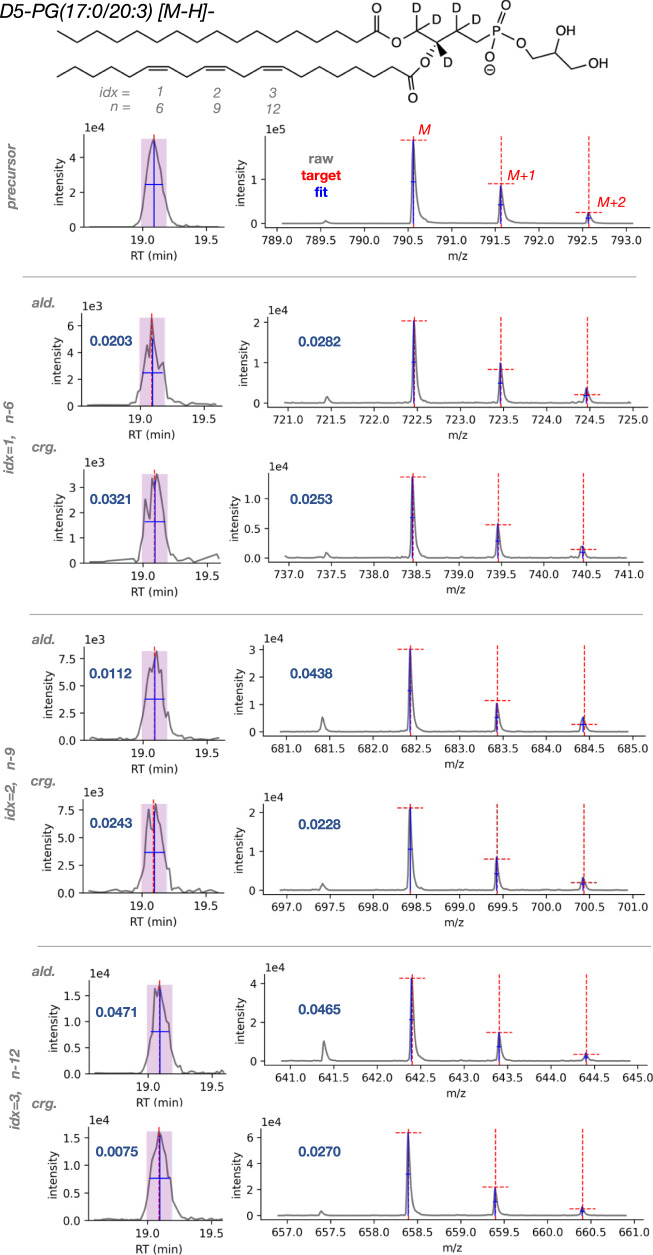
OzID data analysis results for a polyunsaturated lipid standard. Results shown for D5-PG(17:0/20:3) [M-H]^-^ lipid standard, with plots of extracted and processed data for precursor ion and pairs of OzID fragments for each of the 3 double bonds. On the left side of the figure, XICs annotated with fits (blue crosses) and extraction window (shaded region). The right side of the figure shows the RT-selected MS1 spectra with theoretical (red dashed crosses) and observed (blue crosses) isotopic distributions. The chromatographic and isotope distribution scoring components (cosine distance) for all OzID fragments are noted with purple numbers.

A set of lipid standards with known double bond positions (Lipidomix SPLASH and UltimateSPLASH, Avanti Polar Lipids) were initially used to validate the automated data processing workflow in *LipidOz*. LC-MS/MS data of these samples were generated using a Velos Orbitrap mass spectrometer and used to construct the target list consisting of lipid class identity and assign the fatty acyl composition. LC-OzID-IMS-MS data of the same samples were generated using an Agilent 6560 drift tube IMS-QTOF MS platform modified to incorporate the OzID capability for the double bond identification (see following “Methods” section and ref. ^8^ for experimental details). The results for one of these standards in UltimateSPLASH, D5-PG(17:0/20:3), which contains a polyunsaturated fatty acyl chain with double bonds at the *n*-6, *n*-9, and *n*-12 positions, is shown in Fig. 3 as an example for demonstration. The XIC for the precursor ion shows a single clean peak and the retention time (RT)-selected MS1 spectrum contains only the M, M + 1, and M + 2 isotope peaks with masses and abundances that match the theoretically predicted distribution (red dashed lines), supporting the identification of the precursor ion. The next set of plots depict XICs and MS1 spectra for pairs of OzID fragments (aldehyde and criegee with a mass difference of 16 Da), corresponding to each of the three double bonds in this lipid standard. The XICs for all of these OzID fragments display a single peak, matching the retention time of the precursor. The MS1 spectra for these fragments also display M, M + 1, and M + 2 isotope masses and abundances that agree with theoretically predicted isotope distributions, indicating that this set of fragments can be used to confidently assign double bonds at the *n*-6, *n*-9, and *n*-12 positions. Cosine distance was used as a metric to quantify agreement between all fragment XICs versus the precursor, as well as agreement between theoretical and observed isotope distributions (overlaid on XIC and MS1 spectra as purple numbers). All scores for this set of fragments are very low (<0.1), indicating a high degree of similarity between fragment and precursor XICs and likewise for theoretical and observed isotope distributions, supporting the correct assignment of double bond positions for this lipid standard. In contrast, the signals for putative fragments corresponding to other plausible double bond positions from this lipid displayed higher scores with an average of 0.4176 (see Table S1), indicating that their XICs and MS1 spectra do not support assignment of their corresponding double bond positions for this lipid standard. Taken together, these scores quantitatively reinforce that this OzID data supports correct assignment of the known double bond positions from this lipid standard and excludes other plausible double bond positions.

**Fig. 4:**
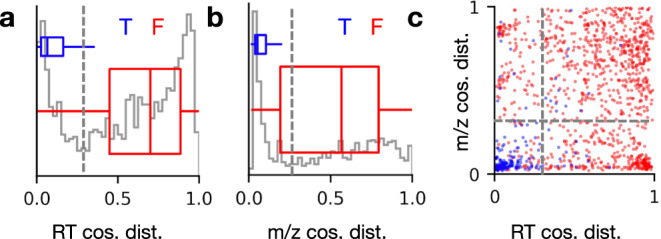
Distributions of chromatographic and mass spectral scoring components for OzID fragments. Distribution of chromatographic (**a**) and mass spectral (**b**) cosine distances from lipid standards and commercial porcine brain total lipid extract data. Gray traces correspond to the total distribution of all examples, while overlaid boxplots (depicting median and interquartile range) show individual distributions for examples assigned as True (blue) or False (red), with decision boundaries as gray dashed lines. **c** Scatter plot of both cosine distances, demonstrating orthogonality between the two scoring components. Individual examples are colored according to whether they are assigned as True (blue) or False (red). The overlaid gray dashed lines indicate the decision boundaries for both dimensions.

In order to develop a more useful heuristic for assignment of double bond positions based on the chromatographic and isotope distribution scoring components described above, we examined the distributions of cosine distances for putative OzID fragments of double bond containing lipids from two sets of deuterium-labeled standards and one well-characterized commercial porcine brain total lipid extract (see Table 1). True examples of OzID fragments consisted of fragment pairs for known double bond positions, while False examples consisted of all putative fragment pairs for all other double bond positions from a precursor that are plausible given its fatty acid composition. Figure 4a shows the distribution of cosine distances corresponding to the similarity between precursor and fragment XICs for OzID fragments from all lipids in this dataset (gray histogram), as well as the individual distributions from True and False examples (blue and red boxplots, respectively). Figure 4b shows the same distributions but corresponding to similarity between theoretical and observed isotope distributions from fragment MS1 spectra. Figure 4c shows a scatter plot of these two scores, with colors indicating True and False examples (blue and red, respectively), demonstrating the orthogonality of these two scoring components. The individual scoring components alone each show clear distinction between known True/False examples (Fig. 4a, b, gray dashed lines), but better discrimination is possible when these components are used in conjunction (Fig. 4c, gray dashed lines). By applying a simple cutoff of 0.25 for both cosine distances (i.e., putative OzID fragments with RT and m/z cosine distances below 0.25 are accepted), we observed 90% accuracy and 15% false discovery rate in assignment of double bond positions for this set of deuterium-labeled standards and porcine brain total lipid extract.

**Table 1:** Summary of lipid data used for training DL model.

	Sample	Replicates	Target Lipids	Lipids Validated	Assigned Double Bond Positions (T/F)
*Positive Ion mode*	SPLASH (SPLA)	1	10	8	8/120
	UltimateSPLASH (ULSP)	2	76	39	68/904
	Brain total lipid extract (BTLE)	1	84	32	91/905
*Negative Ion mode*	SPLASH (SPLA)	4	24	24	136/1810
	UltimateSPLASH (ULSP)	4	140	73	73/1863
				Total	376/5602

For each sample, a proportion of the known target lipid double bond positions were validated, and those validated lipids were used as training examples.

### LipidOz GUI for automated OzID data processing and visualization

**Fig. 5:**
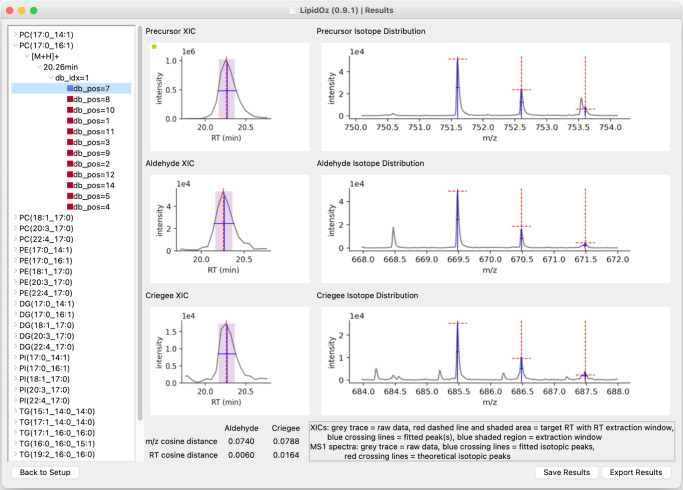
Screenshot of result window in the *LipidOz* GUI application. The Results window allows interactive viewing of OzID analysis results. The panel on the left contains a browsable tree menu with all lipid targets and their putative double bond positions. Each list of putative double bond positions for a single target and double bond index is sorted in descending order of confidence (also reflected by colored squares), as determined by composite score from both corresponding OzID fragments. When a putative double bond position is selected, the panels on the right are populated with plots of the XICs and MS1 spectra for the corresponding precursor and diagnostic pair of aldehyde and criegee fragments, along with all of the individual scoring components. This interface presents all the information necessary for assigning double bond positions from OzID data.

To increase the accessibility of *LipidOz*, a GUI application was developed for setting up OzID data processing and visualizing the results. For ease of use, the application is packaged into an executable file (available for Windows and MacOS) that is bundled with all dependencies and its own Python interpreter. When the application starts, it presents the Setup window (Fig. S1) where the user is prompted to select input data files (in the original instrument format, or in MZA format)^13^ and set data extraction/processing parameters. The user may optionally select an existing results file and proceed directly to viewing the results as well. After setting parameters and pressing the “Process Data” button, the OzID data analysis will proceed, and a different window will appear that displays messages indicating the data processing progress (Fig. S2). When data processing is complete, the user may proceed to the Results window (Fig. 5), where they can interactively browse and view the OzID data analysis and results. There is the option to reannotate incorrect assignments and save the results to a binary format so that they can be viewed later with this application, in addition to the option to export the results into an Excel spreadsheet. This GUI application facilitates analysis of OzID data and makes *LipidOz* accessible to users without Python programming experience.

### Training a deep learning model for assignment of double bond positions from OzID data

**Fig. 6:**
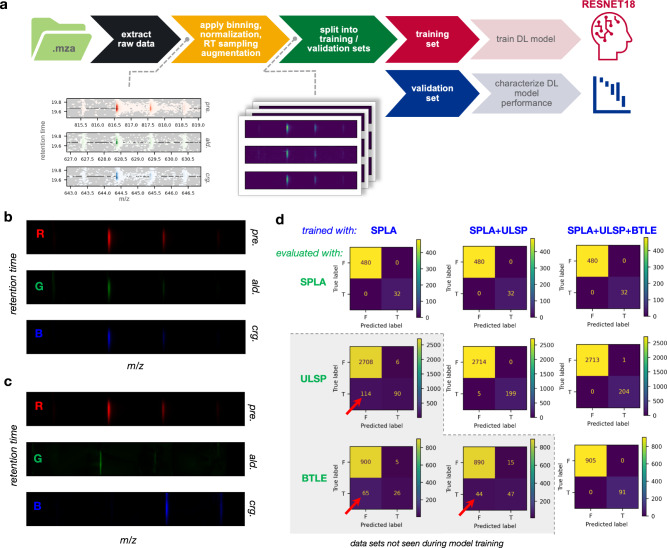
Deep learning-based assignment of double bond positions. **a** DL model training workflow. **b**, **c** 2D LC-MS profiles of precursor, aldehyde, and criegee ions for a True (**b**) and False (**c**) training example. Each LC-MS profile consists of an m/z and retention time dimension, and both groups of profiles are colored in red, green, and blue color scales, reflective of the fact that each training example is constructed as a three-channel RGB image. **d** Confusion matrices demonstrating the classification performance of models trained using different subsets of the data assembled from analysis of lipid standards. The matrices within the gray shaded region correspond to predictions for datasets that were not included during model training, and red arrows highlight false negative classification errors in those comparisons.

The analysis of OzID data described above ultimately boils down to comparisons of 2-dimensional LC-MS profiles between putative OzID fragments and a lipid precursor, with the assignment of double bond position being based upon which pair of putative OzID fragment profiles displays the most “correct” profiles. In this context, a “correct” fragment profile would be one in which the chromatographic component matches that of the precursor and the mass spectral component contains peaks at the appropriate masses and with relative intensities corresponding to the theoretically predicted isotopic distribution. This assessment of fragment profiles can be formulated as a classification task, with the input being LC-MS profiles for a pair of putative OzID fragments and the corresponding precursor, and the output being a Boolean label indicating whether the data for the fragment pair supports assignment of a double bond at that position. Since the input to this classification task consists of three 2D LC-MS profiles (i.e., pseudo-image data), we can treat each profile as a single channel of a RGB image. Figure 6b shows one such RGB image for a set of profiles from a True example, where the precursor profile is assigned to the red channel and the OzID fragment profiles are assigned to the green and blue channels. In these profiles, the m/z component is represented in the x dimension and the retention time component is represented in the y dimension. The m/z values for the M, M + 1, and M + 2 isotopes are aligned across all three channels and are similarly aligned in the center of the y dimension, as expected for a True example. Figure 6c shows a different set of profiles for the same precursor but a different pair of OzID fragments, corresponding to a False example. In this case, poor alignment is observed in both dimensions for the OzID fragment profiles relative to the precursor. Constructing the LC-MS profiles of as RGB images in this fashion decomposes the task of assigning double bond positions from OzID data into a classic image classification task for which there is already a wide variety of deep learning (DL) architectures and models available.

The process of training a deep learning (DL) model for the assignment of double bond locations for lipids from OzID data is consists of two major steps: curation of training data and training/characterizing the model (Fig. 6a). Training a DL model requires a significant amount of labeled training examples, which we constructed from the deuterated lipid standards and well-characterized BTLE (Table 1). We used the automated OzID data analysis workflow in *LipidOz* to extract and process the OzID data for these samples, then manually verified the results and curated a collection of data that could be used for training the DL model. In order to increase the number of examples for training, ~10-fold data augmentation was performed by resampling the RT dimension of the LC-MS profiles in several ways that simulated shifted/narrower/broader chromatographic peaks. Prior to data augmentation, there were 376 True and 5602 False double bond assignments, and after augmentation there were 3024 True and 44816 False examples for DL model training. The DL model was trained with the complete dataset (SPLA + ULSP + BTLE), in addition to two subsets which included either all deuterium-labeled lipid standards (SPLA + ULSP) or only the smaller set of monounsaturated labeled lipid standards (SPLA). The prediction performance of each of these models was assessed with each of the training data subsets in isolation (i.e., SPLA, ULSP, or BTLE), and Fig. 6d contains confusion matrices corresponding to each comparison. Confusion matrices in the section highlighted gray background denote prediction performance for models on individual datasets that were not included during model training. Overall, we found that as long as the subset was present during model training, the DL model was able to achieve nearly 100% prediction accuracy across all examples. When we examine the performance of models on subsets not included during the model training, we begin to see prediction errors, with a distinct bias toward false-negative errors (red arrows in Fig. 6d). Taken together, these results suggest that dl-based double bond assignment can reliably assign double bond positions directly from minimally processed OzID data with high accuracy, provided that the model has seen sufficient representative True examples during training.

### OzID analysis of complex lipid extracts from tissues

**Fig. 7:**
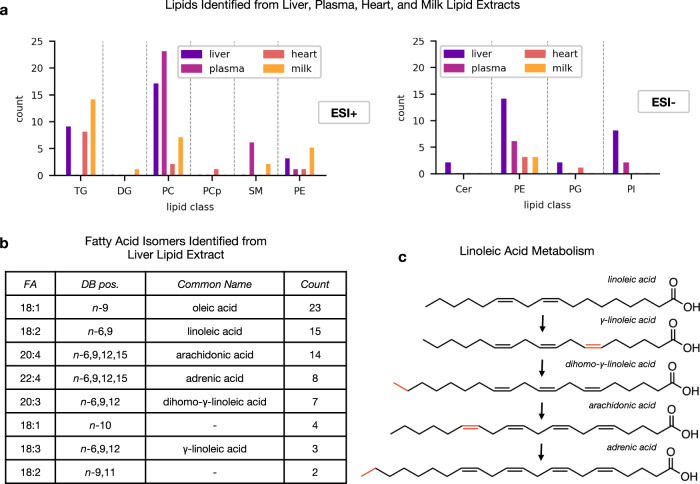
Summary of lipids identified from complex lipid extracts from different tissues. **a** Counts of lipid targets with double bond positions identified from liver, plasma, heart, and milk lipid extracts organized by lipid class, from positive (left) and negative (right) ionization modes. **b** Counts of fatty acid isomers identified in lipid extract from liver. **c** Biosynthetic route from linoleic acid to adrenic acid. The process consists of successive alternating desaturation and elongation steps (highlighted in red).

To demonstrate the utility of *LipidOz* for analysis of OzID data from real samples, we analyzed four complex lipid samples including commercial total lipid extracts from liver and heart (Avanti Polar Lipids, Inc), and NIST SRM 1950 human plasma and SRM 1953 human milk. Initial lipid target lists were constructed from analysis of LC-MS/MS data using LIQUID^12^. The OzID data were analyzed in an automated fashion using *LipidOz* and double bond positions were validated by manual verification. Figure 7a summarizes the counts of lipid targets with identified double bond positions for all tissue extracts, organized by lipid class, in both positive and negative ion modes (the target lists and identified double bond positions for all samples analyzed are summarized and provided as Supplementary Data files: Supplementary Data 1*.xlsx* and Supplementary Data 2*.xlsx*, respectively). At a high level, these results demonstrate that this tool enables detailed characterization of biological samples with broad lipid class coverage in both ionization modes. We also examined the fatty acid isomers that were identified from this analysis to assess the validity of identifications from the *LipidOz* tool. Figure 7b summarizes the counts of fatty acids identified from liver extract. The most common fatty acid identified in this liver sample was oleic acid and components of the biosynthetic pathway between linoleic acid and arachidonic acid^14,15^ (Fig. 6c), which bolsters confidence in the validity of fatty acids identified by OzID data analysis in *LipidOz*. Distinct fatty acid profiles were identified between the different tissue extracts (Fig. S3), further demonstrating the potential biological insights that may be garnered from knowing lipid double bond positions. This demonstration involved processing data from four different complex samples in positive and negative ionization modes with hundreds of lipid targets per run, a scale that would make manual analysis of this complex data practically impossible.

### Observation of sphingolipid backbone fragments

**Fig. 8:**
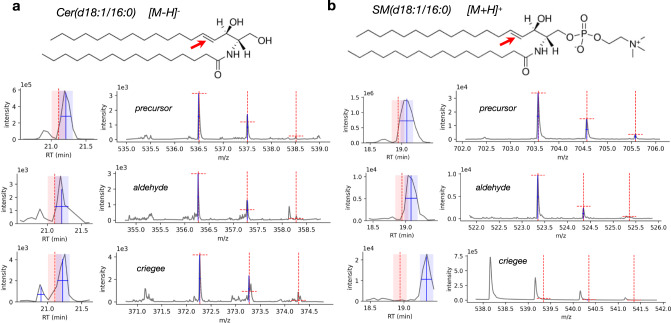
Examples of sphingolipid backbone fragmentation from tissue lipid extract data. **a** Structure of Cer(d18:1/16:0) with backbone double bond (position *n*-14) denoted by red arrow, and corresponding extracted and processed XICs (plots on left side) and MS1 spectra (plots on right side) for precursor and OzID fragments. **b** Structure of SM(d18:1/16:0) with backbone double bond (position *n*-14) denoted by red arrow, and corresponding extracted and processed XICs (plots on left side) and MS1 spectra (plots on right side) for precursor and OzID fragments.

The analysis of tissue lipid extracts also yielded multiple observations of sphingolipid backbone fragments that were not previously observed in OzID studies. Figure 8 shows two examples of these backbone fragments for a ceramide (Fig. 8a) and sphingomyelin (Fig. 8b) species detected in negative and positive ionization modes, respectively. Both the aldehyde and criegee fragments were observed for the ceramide species for double bond position *n*-14, however, the criegee fragment was not observed for the sphingomyelin species, possibly due to presence of an interfering peak and/or low abundance of the expected fragment. Interestingly, among the several examples of sphingomyelin backbone fragments observed in this study, the expected criegee fragments were consistently not observed. This led to the hypothesis that the chemistry of this backbone double bond, specifically the presence of an alpha-OH group, may affect the kinetics of the OzID reaction such that formation of the aldehyde is preferred. This apparent preferential formation of the aldehyde fragment was also observed among the ceramide examples, but less consistently which may indicate the chemistry of the head group having additional influence on OzID kinetics.

## Discussion

In this work we have demonstrated the capability of *LipidOz*, a Python tool, for determination of lipid double bond locations from OzID Data. With this tool, the complex and repetitive OzID data analysis process was automated, allowing for practical application of the OzID technique at scales that are appropriate for routine lipidomics studies. We also demonstrated that application of OzID analysis to larger and more diverse sets of lipids can reveal interesting fundamental insights into the chemistry of the OzID reaction itself. Future experimental studies focusing on the chemistry of OzID would facilitate the application of OzID for double bond assignment in lipidomics research.

*LipidOz* is written in pure Python, with limited and easy to install dependencies for ease of use, and the codebase is open-source so it can be expanded and modified to suit the evolving needs of the community. The flexible Python API in *LipidOz* enables quick and easy scripting while the GUI aids data interpretation and visualization, and together these capabilities facilitate the application of OzID to large-scale biological studies. An important takeaway from this work is the synergy of traditional automation and DL approaches for complex data analysis problems. While the data analysis can be formulated in such a way that is clearly suited to DL, without the traditional automation it would be impractical to process enough data to generate training examples of a sufficient scale for DL.

Interest in the identification of lipid double bonds has increased in recent years, and there have been multiple experimental methods (some with associated software tools) presented in the literature^16–18^. These works are primarily distinguished by the method used to determine double bond locations, specifically, electron-induced dissociation (EID)^16^, oxygen attachment dissociation (OAD)^17^, and Paternò-Büchi(PB) reaction^18,19^. While these techniques all share the underlying principle of producing fragments from lipids with masses that are characteristic to double bond position, the specific analytical configuration (and thus, the structure of the data) as well as the specific fragment masses must be accounted for in software tools in order to support analyzing data from these techniques. *LipidOz* was written specifically to enable automated analysis of OzID data from a LC-OzID-IMS-MS platform, and as such is presently subject to this limitation in scope. However, it is possible that data from these related techniques can be harmonized in such a way that a single tool could analyze data from different analytical platforms and/or methods for determining lipid double bond locations (e.g., UV photodissociation^20^, Paternò-Büchi^19^), which will become increasingly viable as these techniques become more widely used in the field and is an area of ongoing development in *LipidOz*.

In future work, the tool will be expanded to incorporate arrival time and CCS information to provide a greater structural characterization of lipids, as well as identify lipids using LC-IMS-MS/MS such that the lipid target list is generated from same Oz-IMS platform. Support for different experimental methods and analytical platforms can also be accommodated in *LipidOz* with limited modifications to the code, and this is an area of ongoing effort. A particularly impactful future application is the integration of determining double bond positions using OzID with mass spectrometry imaging (MSI) for elucidating the spatial distribution of lipid isomers in tissue sections or other biological systems. MSI has been combined with in situ PB reaction^21,22^, as well as OzID^23,24^, and these combinations have been demonstrated to enable even richer structural characterization of lipids than with MSI alone. Software support for this data is extremely limited and this is an area of focus for development of the *LipidOz* tool.

## Methods

### Sample preparation

The SPLASH and Ultimate SPLASH Lipidomics mixtures of deuterated lipid standards, brain total lipid extract (BTLE, Porcine), heart total lipid extract (Bovine), and liver total lipid extract (Bovine) were purchased from *Avanti Polar Lipids, Inc* (Alabaster, AL). The solvents used in this study, including methanol, chloroform, are LC-MS grade and purchased from Fisher Scientific (Pittsburgh, PA). The deuterated lipid standards were diluted 100-fold in methanol before being subject to MS analysis.

NIST SRM 1950 human plasma and SRM 1953 human milk were quality control samples used for lipidomics research from other projects in our lab. Total lipids were extracted from 50 µL plasma and milk tissue samples using the methyl-tert-butyl ether (MTBE) extraction^25^. Briefly, 55 µL 100% methanol and 185 µL MTBE were added to 50 µL tissue, vortex for 20 s, sonicate on ice for 5 min, incubate on ice for 10 min, vortex for 20 s, and then centrifuge with 15,000×*g* at 4 °C for 10 min. The upper layer containing total lipid extracts were collected, evaporated to dryness in vacuo, and stored at −20 °C in 500 μL of chloroform/methanol (2:1, v/v). Prior to MS analysis, the total lipid extracts were dried down and then reconstituted in chloroform/methanol (1:9, v/v).

### Liquid chromatography-mass spectrometry analysis

LC-MS analysis was performed on a Velos Orbitrap mass spectrometer (Thermo scientific) as previously outlined^12^. For the LC analyses, a Waters Aquity UPLC H class system was used. Standards and extracts were reconstituted in methanol and 10 µL of each were injected onto a reversed phase Waters CSH column (3.0 mm × 150 mm × 1.7 µm particle size). The lipids in the mixture were separated over a 34 min gradient (mobile phase A: acetonitrile/water (40:60) containing 10 mM ammonium acetate; mobile phase B: acetonitrile/isopropyl alcohol (10:90) containing 10 mM ammonium acetate) at a flow rate of 250 µl/min. Eluting lipids were introduced to the MS via electrospray ionization in both positive and negative modes, and lipids were fragmented using higher-energy collision dissociation (HCD) and collision-induced dissociation (CID).

### LC-OzID-IMS-MS analysis

The eluting lipids from the same LC cart and method were analyzed on an Agilent 6560 IMS-MS platform modified to incorporate the OzID technique (LC-OzID-IMS-MS), which was previously described in detail elsewhere^8^ with two additional modifications to improve the efficiency and safety (The information of additional modifications is provided in the Supplementary Information and Fig. S4). Briefly, the instrument was modified to allow introduction of ozone gas (100 g/m^3^) into the high-pressure trapping funnel region before the IMS cell (typical operating pressure of ∼4 Torr). The ions were contained in the trapping ion funnel and allowed to react with the introduced ozone for up to 90 ms prior to injection into the IMS region. The ions exiting the trapping funnel were further separated and analyzed by IMS-MS.

### Lipid nomenclature

The nomenclature for describing lipids used in this manuscript is based on the recommendations of Liebisch et al^26^. As shown in Fig. 1b, the position(s) of unsaturation is indicated to be x carbons from the methyl end of the acyl chain with the nomenclature (*n*-x), different double bonds in polyunsaturated lipids were indicated with index number counting from the end of fatty acyl chain, and the orientation of carbon–carbon double bonds is described as cis (Z) and trans (E) where it is known. For instance, the two double bond locations for the lipid 18: 2 (9Z, 12Z) listed in Fig. 1b is noted as index 1 with position *n*-6 and index 2 with position *n*-9. For lipids containing stable isotope labeling, the degree of labeling is indicated [Dy], where *y* is the number of deuterium atoms.

### Generation of lipid target list

To confirm the lipid class identity and assign the fatty acyl composition of the lipids to generate the target list, lipids were initially identified from LC-MS/MS data using LIQUID^12^. Confident identifications were made by manually evaluating the MS/MS spectra for fragment ions characteristic of the classes and acyl chain compositions of the identified lipids. In addition, the precursor ion isotopic profile, extracted ion chromatogram, and mass measurement error along with the elution time were evaluated. Target lists consisting of the initial lipid identifications and retention times were generated for downstream OzID analysis (Fig. 2a).

### Isotope distribution analysis

Isotope distribution analysis is a standard workflow in *LipidOz* which examines the distribution of M, M + 1, and M + 2 nominal isotopes for putative OzID fragments for determination of double bond position (Fig. 2b). The inputs to this workflow are a list of target lipids (lipid name, ionization state, and retention time), an OzID raw data file (in*. mza* format^13^), and parameters controlling how data is extracted/processed (e.g., tolerances for m/z and retention time). First, an extracted ion chromatogram (XIC) is produced for the precursor m/z in a broad window around the target retention time, and this XIC is fit to obtain the observed retention time which is used for extracting MS1 spectra. An intensity threshold is used to determine whether signal saturation has occurred, and if so a new retention time window is selected for MS1 spectra extraction from the leading edge of the fitted XIC peak^27^. Using the newly determined retention time window, the MS1 spectrum is extracted for an m/z range containing the M, M + 1, and M + 2 nominal isotopes (M-1.5 to M + 2.5 Da), which are each fitted to determine their observed m/z and abundances (peak height). The observed m/z and abundances of the isotopes are compared to theoretical values computed using the molecular formula of the precursor ion. For each lipid target, ranges of possible double bond indices and positions are determined using the lipid’s fatty acid composition. The m/z and molecular formulas for corresponding OzID fragments (aldehyde and criegee) are computed for each combination of double bond index and position, and these are used to extract and fit XICs and MS1 spectra in the same fashion as for the precursor. Finally, a set of scores reflecting the agreement of the chromatographic profiles between precursor and putative OzID fragments in addition to agreement between theoretical and observed isotope distributions for putative OzID fragments are computed.

### OzID fragment scoring

Putative OzID fragments are scored based upon agreement with the precursor chromatographic profile and agreement between its theoretical and observed isotopic distributions. The chromatographic scoring component is obtained by normalizing XICs for the putative fragment and precursor over the same RT range containing the chromatographic peak and computing the cosine distance between them, with a distance of 0 reflecting perfectly overlapping signals and a distance of 1 indicating no overlap (see Fig. S5). The isotope distribution component is computed likewise, but using the RT-selected MS1 spectrum from M-1.5 to M + 2.5 compared against a spectrum constructed from the theoretically predicted isotope distribution based on the fragment’s molecular formula.

### Calculation of theoretical isotope distribution

The theoretical isotope distribution is calculated from the molecular formula using a multinomial expansion method subject to simplifying constraints. Specifically, only heavy isotopes ^13^C, ^15^N, ^18^O, ^33^S, and ^34^S are considered and only M, M + 1, and M + 2 nominal isotope abundances are computed.

### Assignment of double bond location using deep learning

The process of training a deep learning (DL) model for the assignment of double bond locations for lipids from OzID data is consists of two major steps: curation of training data and training/characterizing the model (Fig. 6a). A dataset for DL model training was constructed using the automated OzID data analysis in *LipidOz*. The training data were assembled from two sets of deuterium-labeled lipid standards with known double bond locations and a well-characterized porcine brain lipid extract (Table 1). OzID data analysis was carried out in an automated fashion using *LipidOz* and the results were manually verified prior to data extraction. Data for DL was extracted for each training example as 2D LC-MS profiles (one for the precursor ion and a pair of putative OzID fragments) over the RT ±2.5 min range in the chromatographic dimension and the M−1.5 to M + 2.5 range in the m/z dimension. The sparse scan data in the LC-MS profiles were converted into uniformly sampled image data using 2D linear interpolation, normalized to an intensity range of 0 to 1, and ~10-fold data augmentation was performed by resampling the RT dimension of the LC-MS profiles in several ways that simulated shifted/narrower/broader chromatographic peaks. Finally, the training examples were split into separate training and validation sets in proportions of 4:1, with splitting performed such that the proportion of True/False training examples was maintained across training and validation sets.

A pre-trained RESNET-18^28^ with the terminal fully-connected layers replaced by a 2-node fully-connected layer (one output for True label probability, the other for False label probability) was used as the starting point for model optimization (see Supporting Information for further discussion on model selection). Parameter optimization was done using the Adam optimizer, and cross entropy loss (weighted according to approximate proportions of True/False training examples in training data) was used as the optimization criterion. The model was fed data in batches of 128 training examples and model training was continued over 8 epochs (Fig. S6). The set of parameters yielding minimal loss were saved to file for later use in inference.

### LipidOz implementation

*LipidOz* is implemented in Python and uses standard scientific computing libraries for data processing and visualization (*numpy, scipy, matplotlib*). Raw data extraction is performed using the *mzapy* library (https://github.com/PNNL-m-q/mzapy), which provides utilities for extraction and processing of MS data in the MZA format^13^. *Pytorch* was used for all DL model setup and training. The GUI application was packaged into a standalone executable using *pyinstaller*.

## Supplementary information

Supplementary Information

Description of Additional Supplementary Files

Supplementary Data 1

Supplementary Data 2

## Data Availability

All *LipidOz* code and pre-built GUI executables are available at https://github.com/PNNL-m-q/lipidoz. Documentation including extensive user guides and detailed module-level API documentation is hosted at https://lipidoz.readthedocs.io to support application development and extension of *LipidOz* functionality. Additionally, an example data file for SPLASH lipid standard mixture has been uploaded to 10.5281/zenodo.7636522, which also includes instructions and expected results.

## Data availability

All raw data files generated for this study have been uploaded to MassIVE (https://massive.ucsd.edu/ProteoSAFe/dataset.jsp?accession=MSV000091346). Lipid target lists and identified lipid double bonds information are available in Supplementary Data 1 and Supplementary Data 2 files.
