# Supplementary material for: LipidOz enables automated elucidation of lipid carbon–carbon double bond positions from ozone-induced dissociation mass spectrometry data
Source: Commun Chem. 2023 Apr 19;6:74. doi: 10.1038/s42004-023-00867-9 (PMC10115790; doi:10.1038/s42004-023-00867-9)
Supplement: Supplementary file 1 — Supplementary Information [file 42004_2023_867_MOESM1_ESM.pdf]

Supplementary Information for:

LipidOz Enables Automated Elucidation of Lipid Carbon-Carbon Double Bond Positions from Ozone-Induced Dissociation Mass Spectrometry Lipidomics Data

Dylan H. Ross[1], Joon-Yong Lee[1,2], Aivett Bilbao[1], Daniel J. Orton[1], Josie G Eder[1], Meagan C Burnet[1], Brooke L. Deatherage Kaiser[1], Jennifer E. Kyle[1], Xueyun Zheng[1,*]

1. Pacific Northwest National Laboratory, Richland, WA 99354, USA

2. current address: PrognomiQ, Inc, San Mateo, CA 94403, USA

* To whom correspondence may be addressed, xueyun.zheng@pnnl.gov

Supplementary Discussion

Model Selection for DL-Based Double Bond Assignment

Prior to training the final model presented in this work, we performed smaller scale pilot studies to guide model selection and data curation and processing strategies. For this initial work, we employed the well-known *RandomForestClassifier* implementation in the scikit-learn Python package. This model is particularly effective in dealing with unbalanced small datasets. Data from LipidoMix Splash (SPLA) lipid standards in positive and negative ionization modes was used as the initial model training dataset, which contained a total of 644 training instances (augmented from 8 True and 120 False instances). Our goal was to train the random forest model to determine whether a given double bond position was correct by aligning the isotopic mass profiles based on the theoretical monoisotopic masses of precursor and aldehyde/criegee OzID fragment ions. We divided the data into 70% for model training and 30% for testing. We used 100 estimators in the forest and Gini impurity for split criterion. The test set produced a mean accuracy of 0.96, demonstrating that mass profiles are an effective set of features for building a classifier to identify double bond locations. However, we discovered that mass profiles are insufficient when interfering signals are present. Thus, we introduced the retention time distribution to enhance the accuracy of the model. This expansion into high-dimensional training examples and large image datasets required more complex models, ultimately leading to our selection of RESNET18 as the starting point for training our final DL model using the full sized data set.

Further justification for our model choice comes from our observations while evaluating cosine distances for mass profiles and retention time distributions using our larger set of training examples sourced from analysis of SPLA, ULSP, and BTLE samples. We found that using these cosine distances alone (see Figure 4), we were able to assign double bond positions with an accuracy of 90% and a fairly high FDR of 15% (due to the imbalance of T/F training instances). Consistent with our initial observations, many of the misclassified training examples were due to the presence of interfering signals which arbitrarily shift the cosine distances despite presence or absence of the actual signals of interest. In contrast, the DL model was able to achieve nearly 100% accuracy on the same data, indicating that this more complex model was capable of learning patterns in this complex data in a way that is robust to the presence of interfering signals. Figure S7 shows an illustrative example of a True training instance, which due to the presence of interfering signals has high mass profile and retention time distribution cosine distances, but is correctly classified "True" by the DL model.

Supplementary Methods

Experimental setup for LC-OzID-IMS-MS Analysis

The eluting lipids from LC were analyzed on an Agilent 6560 IMS-MS platform (Agilent; Santa Clara, CA) modified to incorporate the OzID technique (LC-OzID-IMS-MS), which was previously described in detail elsewhere(Poad, Zheng et al. 2018). The ozone gas was generated from pure oxygen using the ozone generator HG-1500 (Ozone Solutions; Sioux City, IA). The ozone was introduced to the trapping region of the IMS-MS like the setup from the previous study(Poad, Zheng et al. 2018), with two modifications (highlighted in orange circles the Figure S4). First, between the destructor and the tee that connects it to the ozone monitor a needle valve was added to control the amount of ozone going to waste. Second, the PEEK line that introduces the ozone, nitrogen mixture to the trapping funnel was extended into the instrument further and directed the gas mixture directly into the path of the ions. With the lower flowrate the ozone generation yields a concentration of about 100 g m^{-3} at an oxygen flowrate of 0.08 L min^{-1}. This directed flow resulted in similar double bond fragmentation and is more reproducible. Also, with less ozone going to the destructor the oxygen supply can easily last through hundreds of samples, while not compromising on safety.

Table S1: Scores for D5-PG(17:0/20:3) [M-H]- Putative OzID Fragments. RT and m/z cosine distances for all putative OzID fragments from D5-PG(17:0/20:3). Highlighted rows reflect the assigned double bond positions.

		(aldehyde)		(criegee)	
DB idx	DB pos	RT cos. dist.	m/z cos. dist.	RT cos. dist.	m/z cos. dist.
1	6	0.0203	0.0282	0.0321	0.0253
	9	0.0463	0.6439	not found	
	1	0.3421	0.8538	not found	
2	9	0.0112	0.0438	0.0243	0.0228
	10	0.4830	0.0283	0.7493	0.5070
	12	0.0267	0.5173	not found	
	13	not found		0.2533	0.7755
3	12	0.0471	0.0465	0.0075	0.0270
	8	0.0513	0.5689	not found	

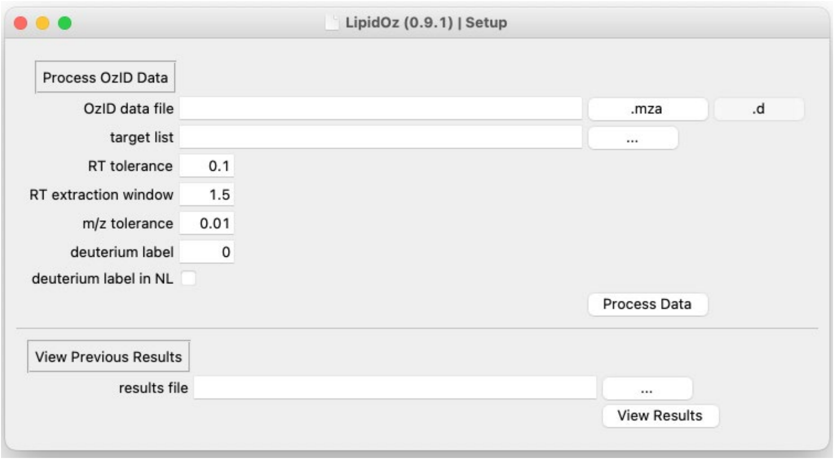

Figure S1: Screenshot of LipidOz GUI application setup window

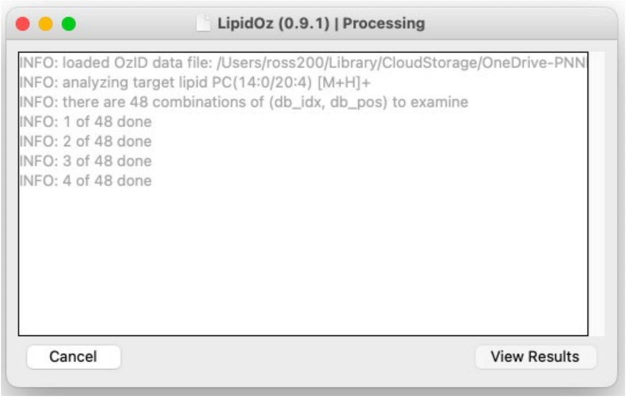

Figure S2: Screenshot of LipidOz GUI application processing window

plasma

FA	DB pos.	count
18:1	*n*-9 (oleic acid)	7
20:4	*n*-6,9,12,15 (arachidonic acid)	4
18:2	*n*-6,9 (linoleic acid)	4
16:1	*n*-7 (palmitoleic acid)	4
20:3	*n*-6,9,12 (dihomo-γ-linolenic acid)	3
18:1	*n*-7	2
20:4	*n*-3,6,9,12	2

liver

FA	DB pos.	count
18:1	*n*-9 (oleic acid)	16
20:4	*n*-6,9,12,15 (arachidonic acid)	10
18:2	*n*-6,9 (linoleic acid)	9
22:4	*n*-6,9,12,15 (adrenic acid)	5
20:3	*n*-6,9,12 (dihomo-γ-linolenic acid)	4
18:1	*n*-10	4
18:2	*n*-9,11	2
18:3	*n*-6,9,12 (γ-linolenic acid)	2

heart

FA	DB pos.	count
18:1	*n*-9 (oleic acid)	6
18:2	*n*-6,9 (linoleic acid)	2

milk

FA	DB pos.	count
18:1	*n*-9 (oleic acid)	12
18:2	*n*-6,9 (linoleic acid)	11
18:2	*n*-9,11	2
18:1	*n*-6	2

Figure S3: Counts of Fatty Acids Identified from Tissue Extracts, including total lipid extracts from liver and heart, and NIST SRM 1950 human plasma and SRM 1953 human milk.

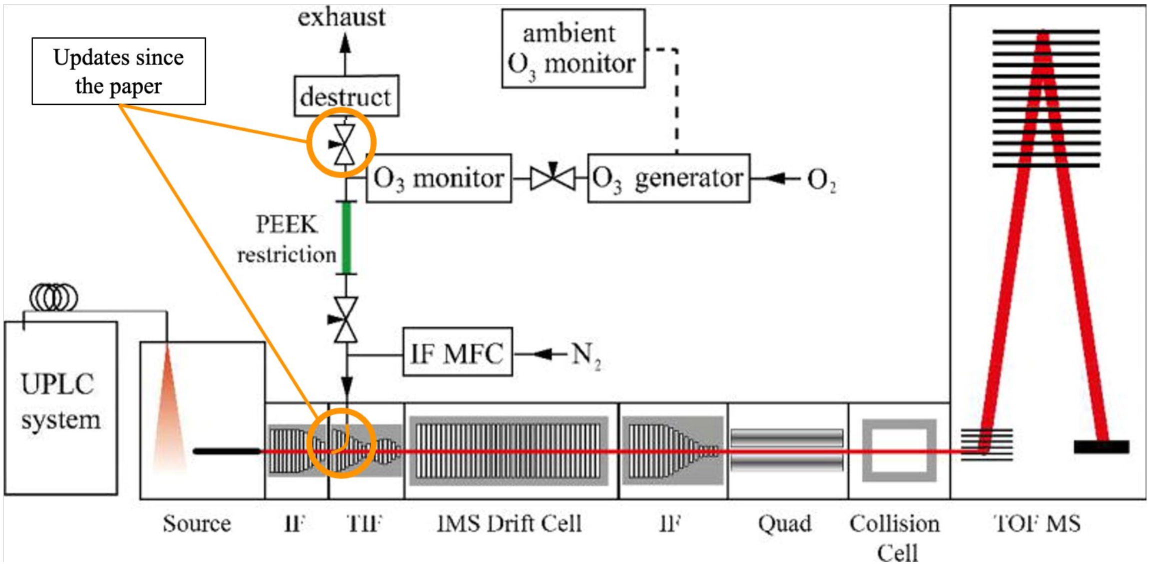

Figure S4: Schematic overview for introduction of ozone into the high-pressure trapping ion funnel of the Agilent 6560 IMS q-TOF mass spectrometer. Ozone was introduced to the N_2 line after the ion funnel mass flow controller (IF MFC). The two additional modifications after previous study were highlighted in orange circles. The figure was adapted with permission from Poad, B. L. J., et al. (2018).[1] "Online Ozonolysis Combined with Ion Mobility-Mass Spectrometry Provides a New Platform for Lipid Isomer Analyses." Analytical Chemistry **90** (2): 1292-1300. Copyright © 2018 American Chemical Society."

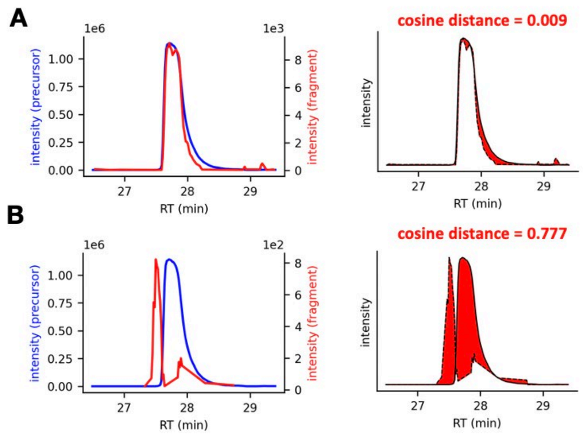

Figure S5: Demonstration of Cosine Distance Scoring. (**A**) Example of precursor (blue) and putative fragment (red) XICs with good overlap. The cosine distance reflects the non-overlapping area (red shaded area) between the two signals, which is very small due to the high degree of overlap. (**B**) Example of XICs with poor overlap. The red area (and cosine distance) is relatively large, reflecting

the poor overlap.

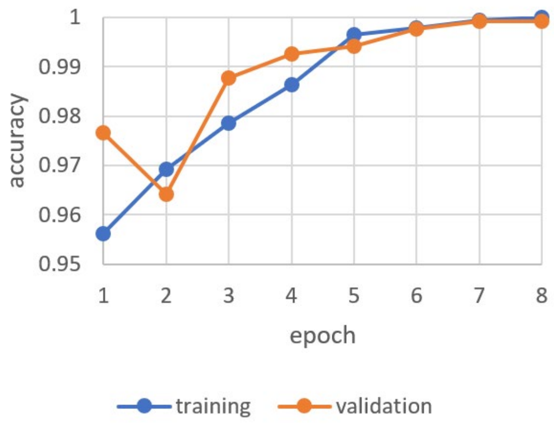

Figure S6: Training/Validation Accuracy During DL Model Training. Accuracy scores for training and validation sets during 8 epochs of DL model training using the combined SPLA + ULSP + BTLE data set were plotted.

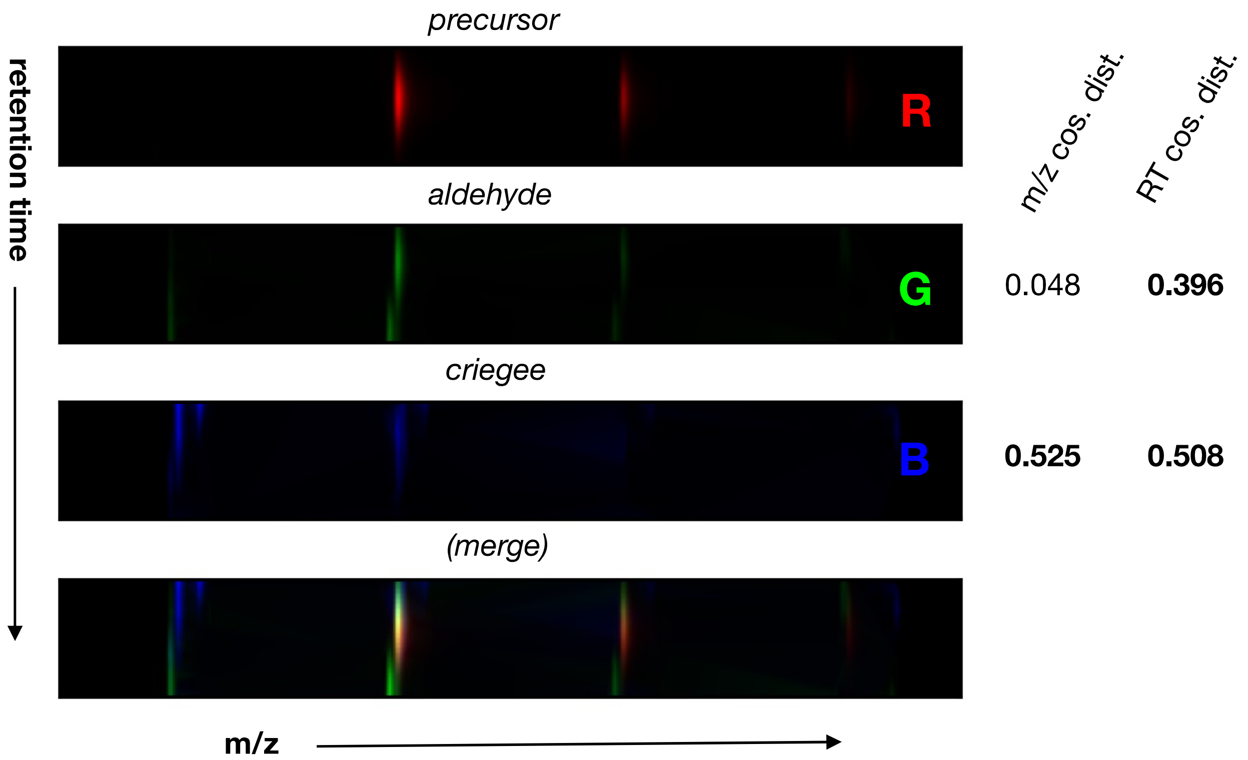

Figure S7: Example of a True training instance from BTLE which contains interfering signals. The interfering signals lead to high cosine distances for both fragments, which if used as the basis for classification would lead to misclassification of this instance. The DL model correctly assigns this training instance as True.

Supplementary References:

1. Poad, B. L. J., et al. (2018). "Online Ozonolysis Combined with Ion Mobility-Mass Spectrometry Provides a New Platform for Lipid Isomer Analyses." Analytical Chemistry **90**(2): 1292-1300.
