# Supplementary material for: LipidOz enables automated elucidation of lipid carbon–carbon double bond positions from ozone-induced dissociation mass spectrometry data
Source: Commun Chem. 2023 Apr 19;6:74. doi: 10.1038/s42004-023-00867-9 (PMC10115790; doi:10.1038/s42004-023-00867-9)
Supplement: Supplementary file 2 — Description of Additional Supplementary Files [file 42004_2023_867_MOESM2_ESM.pdf]

Description of Additional Supplementary Files

File name: Supplementary Data 1

Description: Lipid target lists for all the samples analyzed in this work.

File name: Supplementary Data 2

Description: Identified lipid double bonds for all the samples analyzed in this work.
